# Use of Dorsal Ulnar Artery Flap for Coverage of Arterio-Cutaneous Fistula over Post-Electrical Burn Scar: A Case Report

**DOI:** 10.29252/wjps.9.2.238

**Published:** 2020-05

**Authors:** Rishabh Joshi, Deepak Krishna, Manal M. Khan

**Affiliations:** Department of Burns and Plastic Surgery, AIIMS, Bhopal (M.P.), India

**Keywords:** Dorsal ulnar artery flap, Arterio-cutaneous fistula, Electrical burn, Scar

## Abstract

We reported a 38 year old male patient who suffered from electric burn 2 years ago, and came with complaints of recurrent profuse bleeding from post electric burn scar over left wrist area since last 6-8 months. We successfully used the dorsal ulnar artery flap to cover the arterio-cutaneous fistula over the post-electrical burn scar.

## INTRODUCTION

Arterio-cutaneous fistula of radial artery is extremely rare complication in post-electrical burn scars of the upper limb. Reconstruction of the soft tissue defect in the hand and wrist has always been challenging. Durable and stable coverage of soft tissue defects in the hand and wrist with a thin, pliable and large enough cutaneous flap (local or regional) seems to be an ideal solution. The dorsal ulnar artery fasciocutaneous flap was first described in 1988, perfused by the ascending branch of the dorsal ulnar artery, one of the major branches of the ulnar artery in the distal forearm.^1^

The best advantage of this flap lies in the possibility of mobilization of tissue without losing a major vascular axis. The dorsal ulnar artery fasciocutaneous flap can be raised as a hinge (peninsular), or as a true island flap.^2^ Here, we are presenting a case report, where the dorsal ulnar artery flap was used to cover the arterio-cutaneous fistula over the post electrical burn scar in a 38 years old male over the left wrist.

## CASE REPORT

A 38 year old male patient who suffered from electrical burn 2 years before, came with complaints of recurrent profuse bleeding from post-electrical burn scar over the left wrist area since last 6-8 months. Distal hand function was normal. There was no sensory loss. Perfusion of the hand was also normal. On color Doppler assessment, there was normal flow in both the ulnar and the radial artery with a possible defect in distal part of the radial artery underneath the post-electrical burn scar. A written consent was provided from the patient.

**Fig. 1:**
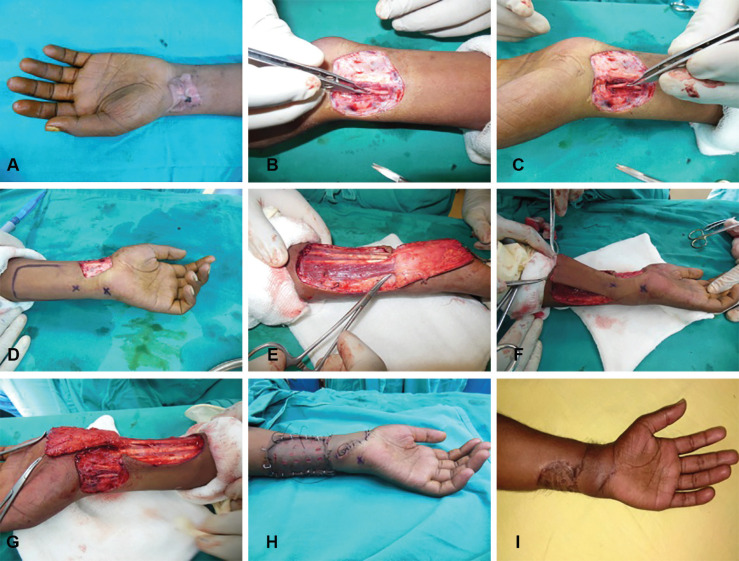
**A-I: **The scar treated over the left wrist

The scar over the left wrist was marked (Figure 1A) and excised with exploration under tourniquet. A partial defect was found in the radial artery with distal lumen continuity which was the source of bleeding (Figure 1B and 1C). The radial artery fistula was repaired and distal flow was checked. The origin of dorsal ulnar artery was marked with hand held Doppler (Figure 1D and 1E) as 6x6 cm sized flap that was raised and dissected up to the origin of dorsal ulnar artery (Figure 1E and 1F). The flap was transposed over the defect. The donor area was covered with SSG (Figure 1G and 1H). Flap was healthy during follow up period.

## DISCUSSION

Arteriocutaneous fistula has been rarely reported with complication, mostly in procedures involving major vessels bypass graft or aneurysm repair surgeries.^3^^,^^4^ It has not been mentioned in post-electrical burn scar in the literature till now. Achieving suitable flap coverage for post-electrical burn or post-surgical defects following scar excision around the wrist and hand has always been a challenge. The perforator based flaps of ulnar or radial artery are preferred option for coverage in wrist and hand defects.^3^^,^^4^

Especially in cases where one of the major vessels of the hand is involved due to trauma or electrical burn; thus only limited options are available. If both the major vessels of the hand are intact, then either one vessel can be used in pedicled flap or as recipient vessel for free flap. Other preferred options include posterior interosseous artery (PIA) flap, which does not compromise the major vessels; but it is a time consuming procedure and needs more expertise in comparison to perforator based flaps of radial or ulnar artery.^2^^,^^5^

Distal flaps (e.g. groin flap) result in immobilization of the hand for long periods. Free flaps require microsurgical expertise and involve a prolonged operative procedure with sophisticated postoperative care. In this case, dorsal ulnar artery (DUA) flap was the preferred option as none of the major vessel could be compromised as radial artery was involved in the arterio-cutaneous fistula. Also DUA flap was locally available. 

## CONFLICT OF INTEREST

The authors declare no conflict of interest.
